# Comparative analysis of interactions between aryl hydrocarbon receptor ligand binding domain with its ligands: a computational study

**DOI:** 10.1186/s12900-018-0095-2

**Published:** 2018-12-06

**Authors:** Kumaraswamy Naidu Chitrala, Xiaoming Yang, Prakash Nagarkatti, Mitzi Nagarkatti

**Affiliations:** Department of Pathology, Microbiology and Immunology, University of South Carolina, School of Medicine, Columbia, SC 29208 USA

**Keywords:** Aryl hydrocarbon receptor, Molecular modelling, Molecular dynamic simulations, Dietary compounds

## Abstract

**Background:**

Aryl hydrocarbon receptor (AhR) ligands may act as potential carcinogens or anti-tumor agents. Understanding how some of the residues in AhR ligand binding domain (AhRLBD) modulate their interactions with ligands would be useful in assessing their divergent roles including toxic and beneficial effects. To this end, we have analysed the nature of AhRLBD interactions with 2,3,7,8-tetrachlorodibenzo-ρ-dioxin (TCDD), 6-formylindolo[3,2-b]carbazole (FICZ), indole-3-carbinol (I3C) and its degradation product, 3,3′-diindolylmethane (DIM), Resveratrol (RES) and its analogue, Piceatannol (PTL) using molecular modeling approach followed by molecular dynamic simulations.

**Results:**

Results showed that each of the AhR ligands, TCDD, FICZ, I3C, DIM, RES and PTL affect the local and global conformations of AhRLBD.

**Conclusion:**

The data presented in this study provide a structural understanding of AhR with its ligands and set the basis for its functions in several pathways and their related diseases.

**Electronic supplementary material:**

The online version of this article (10.1186/s12900-018-0095-2) contains supplementary material, which is available to authorized users.

## Background

The aryl hydrocarbon receptor (AhR) is a widely expressed heterodimeric transcriptional regulator, belonging to the basic helix-loop-helix family, in mammals. AhR plays a prominent role in the mechanistic facilitation of biotransformation and toxicity elimination encountered from the environment [1]. This receptor is a transcription factor inducing the expression of a large number of genes and producing different biological and toxic effects [2]. AhR is distinct from other members of the Per-Arnt-Sim (PAS) proteins by being able to be activated with ligands [3] such as 2,3,7,8-Tetrachlorodibenzo-p-dioxin (TCDD), 6-formylindolo[3,2-b]carbazole (FICZ), kynurenine and 2-(1′H-indole-3′-carbonyl)-thiazole-4-carboxylic acid methyl ester (ITE) [4], Indole-3-carbinol (I3C) [5], Diindolylmethane (DIM) [6], Resveratrol (RES) [7] and the like, known to mediate cellular responses to such ligands and metabolic responses to the toxic compounds.

AhR also plays a critical role in regulating the functions of immune, hepatic, vascular, cardiovascular and reproductive systems [8]. AhR activation has been implicated in immune responses specifically in the differentiation of T regulatory cells (Tregs) as well as T helper (Th)-17 cells [9]. Previous studies also showed that AhR controls IL-19 and IL-22 production thereby regulating T cell differentiation and consequently autoimmune diseases and immune pathology [10, 11]. AhR is vital for the dendritic epidermal γδ T-cell maintenance and tissue-resident memory T cell persistence in the skin [12].

Recent reports showed that AhR regulates the differentiation of Th17 and Tregs in a ligand-specific manner [10, 11, 13] and the major factors affecting the outcome of gene transcriptional regulation by AhR include i) nature and affinity of the ligand ii) the specific cell type and co-activators in the cells expressing AhR [14]. AhR is known to be activated by numerous ligands including environmental pollutants such as TCDD, plant products such as I3C, DIM, and RES which have been shown to promote the differentiation of CD4 + Foxp3+ Tregs and inhibit the Th17 cells [15]. In contrast, FICZ, which is an endogenously produced AhR ligand, produces the opposite effect by inducing Th17 cells and downregulating Foxp3+ Tregs [16]. Despite the importance of AhR activity in regulating these differential effects, the precise mechanism or interactions underlying its activity regulation with these ligands, remain poorly understood. Recent studies from our lab demonstrated that this may result from the ability of AhR ligands to induce differential expression of microRNA [17].

**Fig. 1:**
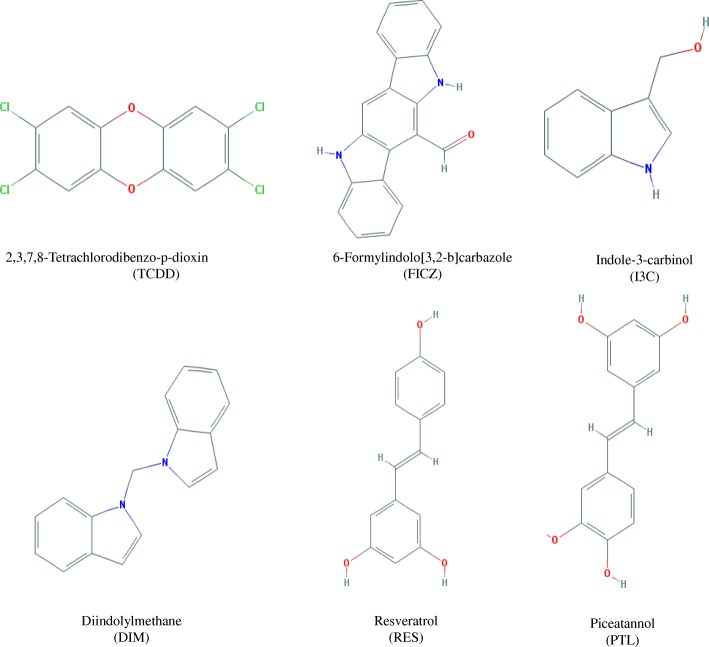
Chemical structures of the ligands used in the study. In the present study, the effect of AhR ligands (TCDD, I3C, FICZ, DIM, RES, PTL) on the protein dynamics, structure, stability and free energy landscape was examined using MDS and free energy calculations. Specifically, different conformational and ligand bound states of AhRLBD-TCDD, AhRLBD-FICZ, AhRLBD-RES, AhRLBD-PTL, AhRLBD-DIM, AhRLBD-I3C complexes were simulated to reveal the entire catalytic cycle, including the conformational change, substrate binding, protein dynamics, side chain interactions, and thermodynamics. The study provides a molecular level understanding of how the residues on AhR LBD interact with these ligands

The interactions between AhR and its ligand may depend on dynamical properties which may not be evident from a single static structure. Despite recent advances in crystallography, the complete three-dimensional structure for AhR is ill defined and therefore a limited amount of information is available regarding the specific interactions of the different ligands with AhR. Recent studies showed only the crystal structure of AhR in complex with the other receptors [18–20]. Several studies demonstrated that AhR ligands modulate the inflammatory response in different ways [21–29]. To better understand the interactions between AhR and its ligands herein, using AhR Ligand binding domain (LBD) three dimensional structure, the mechanism of TCDD (a herbicide used in the Vietnam War), FICZ (a degradation product of tryptophan), Resveratrol (RES, a polyphenol present in the red grapes, peanuts and berries), Piceatannol (PTL, an analog of RES), Indole-3-carbinol (I3C, present in broccoli, cabbage, cauliflower, brussels sprouts, collard greens, kale) and 3,3′-diindolylmethane (DIM, derived from the digestion of I3C present in cruciferous vegetables) regulation/interactions by its binding residues was investigated through molecular dynamic simulations (MDS) and binding free energy calculations (Fig. 1). Such simulations are previously shown to capture the complete process of ligand or drug binding to the receptor, with the ligand exploring a receptor’s surface and ‘discovering’ the binding conformation at crystallographic accuracy, without knowledge of the binding site [30, 31] for ligands.

## Results

### AhR LBD structure

**Fig. 2:**
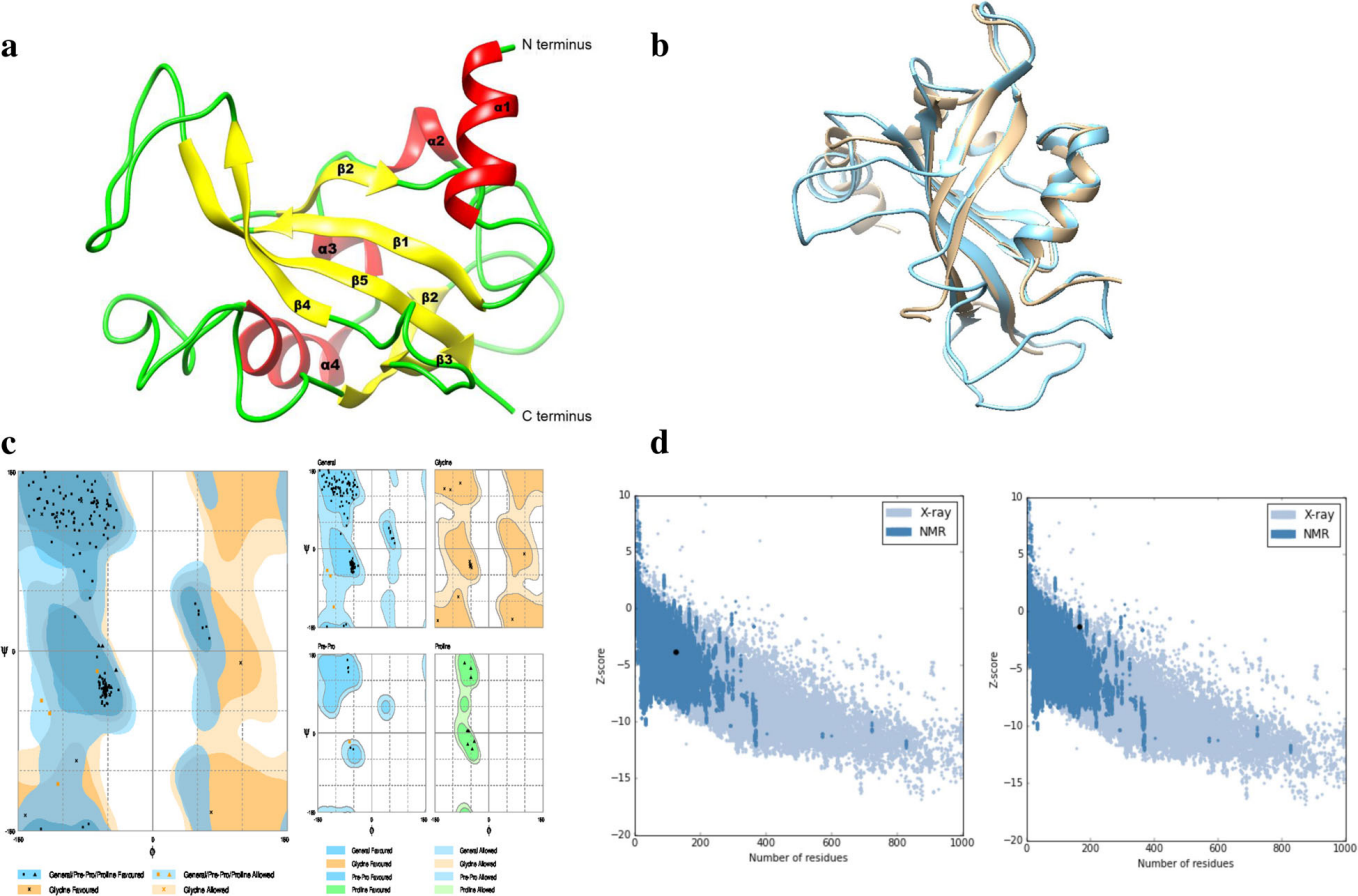
Homology model and Quality metrics of AhR LBD. **a** Modelled structure of mouse AhR LBD with helices are shown in red, sheets are shown in yellow and loops are shown in green color. The figure indicates the vicinity of the α1-helix to the N-terminus. **b** Superimposition of the template (light blue) with the mouse AhR LBD model (light brown) in cartoon secondary structure with an RMSD 1.02 Å using CLICK server. **c** Ramachandran plot showing energetically allowed regions for backbone dihedral angles ψ against ϕ of amino acid residues in modelled mouse AhR LBD protein structure. The plot of AhR LBD model shows 97.6% residues in favored region, 2.4% residues in allowed region and 0.0% residues in outlier region from the total residues. **d** Represents the ProSA analyses of the generated mouse AhR LBD structure model. The calculated quality (Z) scores (closed circles) are displayed in the context of all experimentally determined protein structures available in the Protein Data Bank with each dot representing a distinct structure solved by X-ray crystallography (light blue) or NMR (dark blue). The Left side of the figure represents the prosa-web plot of template 4M4X chain A with a z-score value of − 3.86 whereas the right side of the figure represents the prosa-web plot of built AhR LDB model with a z-score value of − 1.37

To date, the three-dimensional (3D) structure for mouse AhR LBD is unavailable. Only the crystallographic structure for AhR PAS-A domain (PDB ID: 4m4x) [32] and AhR-ARNT (AhR nuclear translocator) heterodimer in complex with the dioxin response element (DRE) (PDB ID: 5v0l) [18] is available in the PDB database. Therefore, we constructed the 3D model for AhR LBD using homology modelling followed by the validation using ProSA (Protein Structure Analysis) web and Rampage web servers. Results from the validation of built 3D model using Ramachandran plot from Rampage web server showed that 97.6% of the total residues were in the favoured region, 2.4% of the residues in the allowed region, and none of the residues were located in the disallowed region confirming that the protein backbone dihedral angles phi (Φ) and psi (Ψ) occupied reasonably accurate positions in the built 3D model (Fig. 2c). Results from the ProSA analysis showed the z-score for the 3D model within the range of scores typically found for native proteins of similar size (Fig. 2d). Further, results from superimposing the built model with template structure (4M4X-A) using CLICK server [33] and chimera 1.10.1 showed an RMSD less than 1.5 Å and a structure overlap of 90.48% indicating that the built model has a similar symmetry to its template structure (Fig. 2b). Results from the analysis of bad contacts, bond lengths, bond angles, Φ and Ψ angles from Ramachandran plot and RMSD predictions from CLICK server concluded that the generated structure model (Fig. 2a) for AhR LBD is reliable for further studies.

### Binding pocket for mouse AhR LBD

**Fig. 3:**
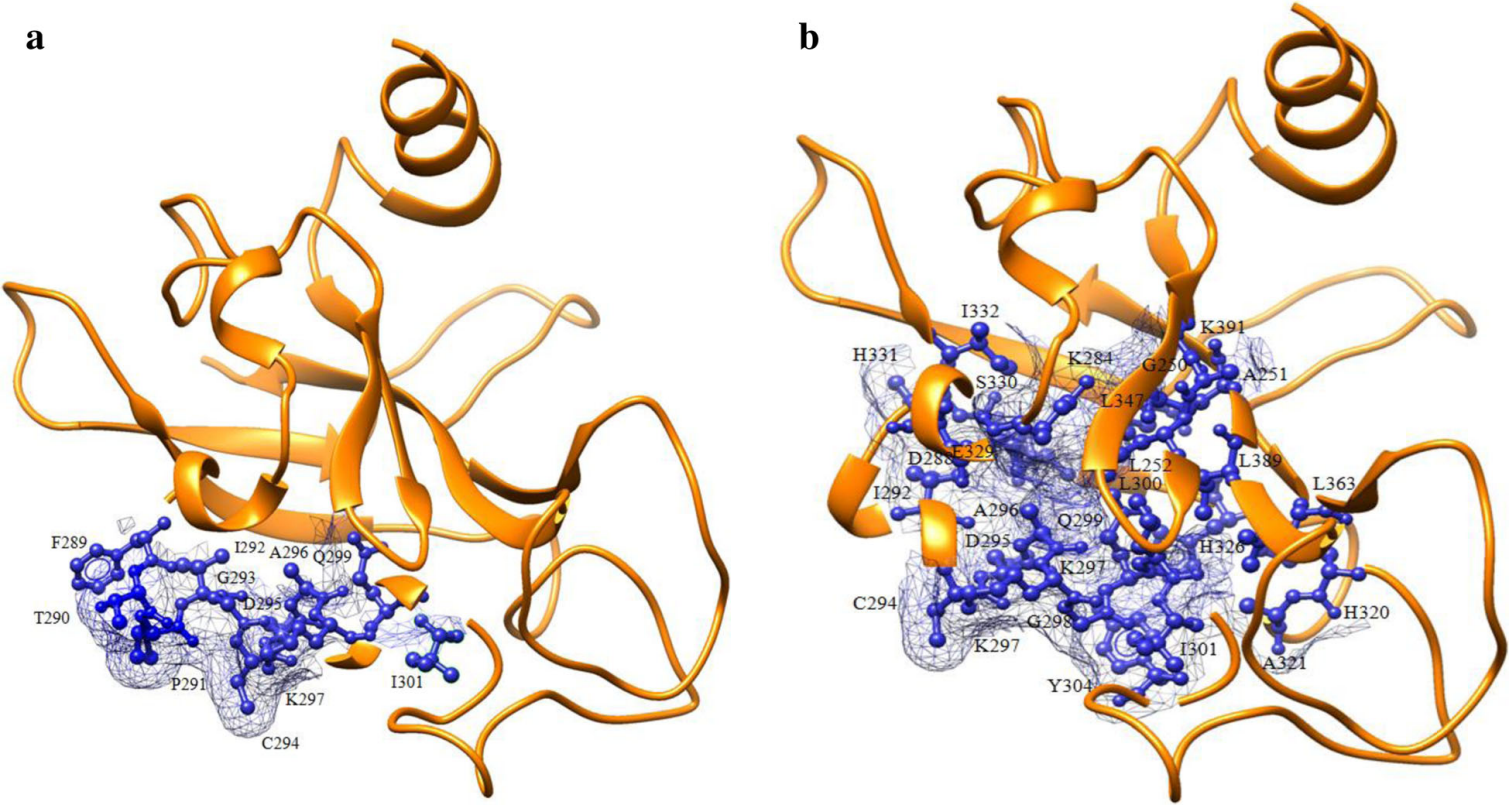
Binding pockets predicted for AhR LBD. **a** Represent the binding pocket predicted using structure-based alignment method **b** Represent the binding pocket predicted using 3DLigandsite server. AhR LBD is represented in orange color ribbons; binding pocket residues are represented in medium blue color ball and sticks with wire surface representation

Results from the 3D- BLAST search for structure-based alignment binding pocket prediction method showed 20 PDB structures that are homologous to the mouse AhR LBD (see Additional file 1). Among these structural hits, crystal structure of the photoactive yellow protein mutant T50 V (PDB ID: 1f98 chain A) and structure of the redox sensor domain of *Methylococcus capsulatus* (Bath) MmoS (PDB ID: 3ewk chain A), which have least identity percentage and high e-value (PBD ID: 1f98 and 3ewk) were not considered for further analysis. Results from the alignment of structural homologs using the Mulitprot server showed the residues Phe289, Thr290, Pro291, Ile292, Gly293, Cys294, Asp295, Ala296, Lys297, Gln299, Ile301 (Fig. 3a) forming the binding site with an optimal RMSD of 1.12 Å and a core alignment size of 11. Results from the Lesk-Hubbard plot displayed number of residues within the structures with the RMSD from the Mustang server (Additional File 2 C). Results from the 3DLigandSite binding pocket method showed the residues Gly250, Ala251, Leu252, Lys284, Asp288, Ile292, Cys294, Asp295, Ala296, Lys297, Gly298, Gln299, Leu300, Ile301, Tyr304, His320, Ala321, His326, Glu329, Ser330, His331, Ile332, Leu347, Leu363, Leu389, Lys391 (Fig. 3b) forming the binding pocket with a cluster of 30 ligands having an average MAMMOTH score of 9.2. These binding site residues are in accordance with the previously reported AhR LBD binding cavity residues that are predicted using site-directed mutagenesis experiments [34, 35].

### Analysis of AhR ligands binding to AhR LBD

**Fig. 4:**
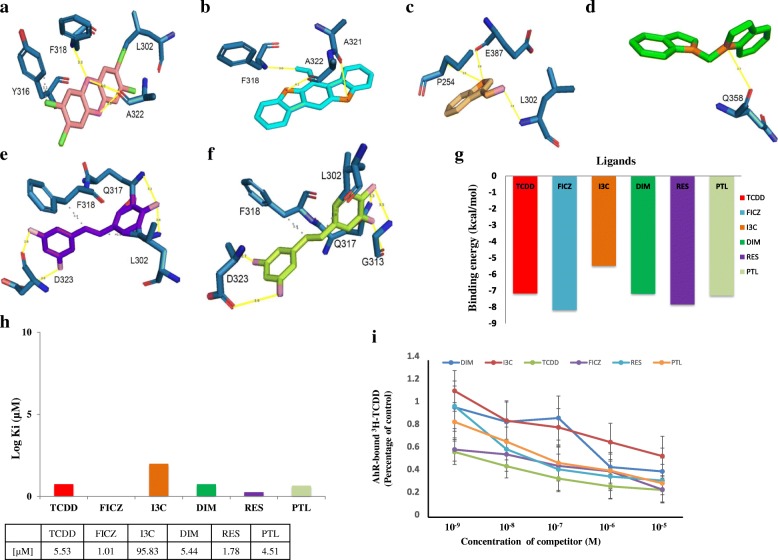
Protein-ligand interactions across the binding pocket of AhR LBD. **a, b, d, e, f** Represent the docked poses of AhRLBD with its ligands at the residues predicted using blind docking approach (**c**) Represent the docked poses of the AhRLBD with its ligands at the residues predicted using Ligsite server. TCDD is shown in salmon red color sticks, FICZ is shown in cyan color sticks, I3C is shown in light orange color sticks, DIM is shown in green color sticks, RES is shown in purple color sticks, PTL is shown in limon color sticks. AhR LBD residues are shown in sky blue color sticks. Hydrogen bonding interactions were shown in yellow color and hydrophobic interaction were shown in grey color lines. **g** Represent the bar charts showing the binding energies of each ligand against AhRLBD. **h** Represent the bar charts showing the log 10 estimated inhibition constants (Ki) by Autodock for each ligand against AhRLBD. **i** Competitive binding assay of AhR ligands with the AhR-bound ^3^H-TCDD

**Fig. 5:**
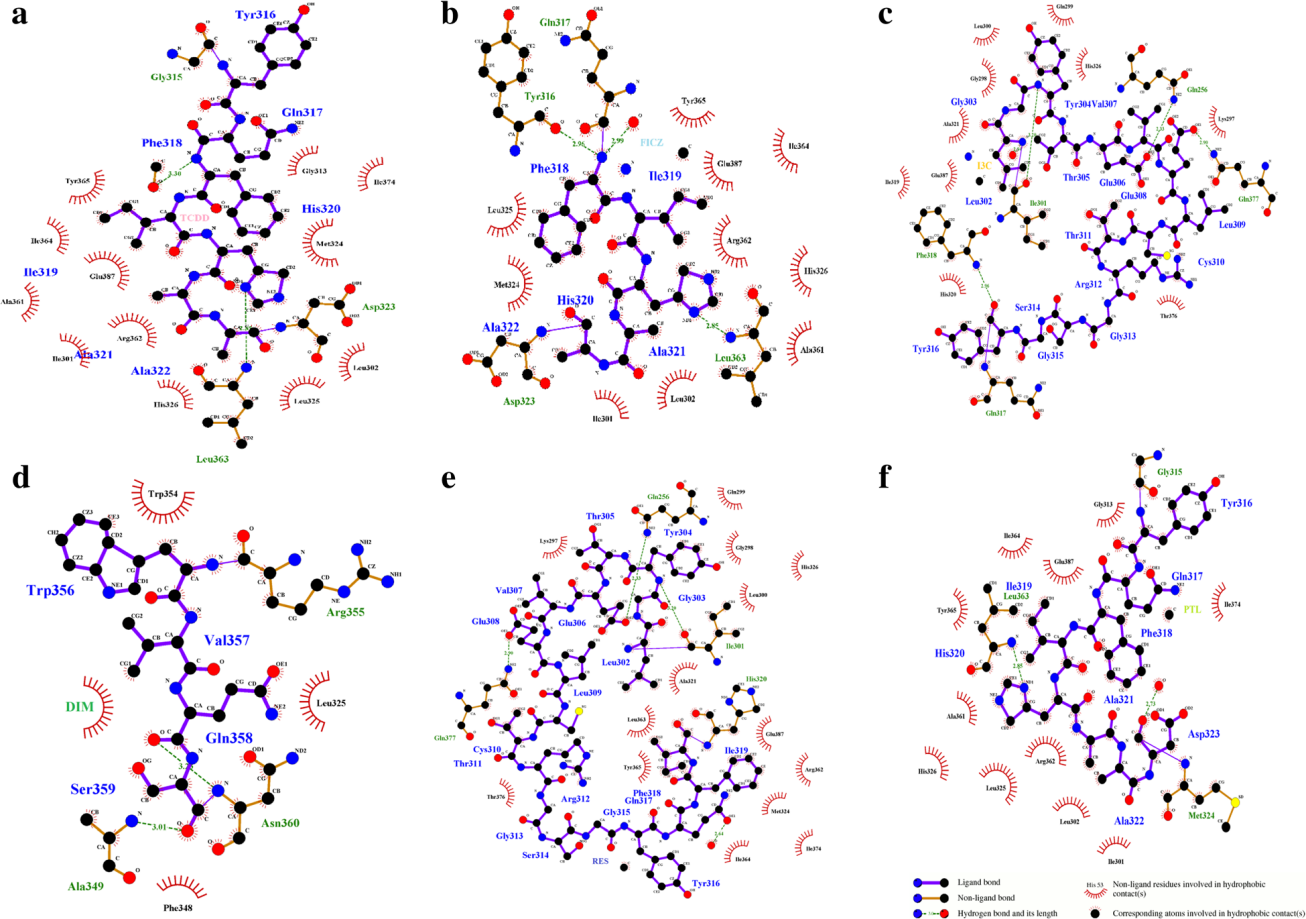
Ligplot analysis of AhRLBD-ligand interactions shown in the Fig. 4. Ligplot showing the interactions of AhR LBD with **a** TCDD **b** FICZ **c** I3C **d** DIM **e** RES **f** PTL. Green lines indicate the hydrogen bonds and red dotted lines indicate the hydrophobic interactions

To explore the binding modes of each ligand, molecular docking was carried out for each ligand at the AhR LBD binding sites predicted by the structure-based alignment and 3DLigandSite server methods and the results were compared with the molecular docking results from the blind docking approach (Additional file 3 A-I). Results showed that the ligands, TCDD, FICZ, DIM, RES, and PTL have high-affinity poses for blind docking approach whereas I3C has high-affinity pose for the 3DLigandSite approach (Table 1) (Fig. 4g and h). Docking results indicated that these compounds can bind tightly in these binding sites. The respective docking poses at each binding site was shown in the Fig. 4a-f and Additional file 3 A-L. Therefore, the high affinity poses shown in the Fig. 4a-f were selected for further study. The summary of interacting amino acid residues of AhR LBD with the various AhR ligands at each of these positions was shown (See additional file 4). Validation of these results using the competitive binding assay with AhR bound ^3^H-TCDD showed a similar pattern with FICZ having the highest affinity and I3C with least affinity towards AhR (Fig. 4i). Overall, these results showed that FICZ has high affinity towards AhRLBD compared to the other ligands (Table 1). Further, ligplot analysis showed that each of these ligands bind perfectly at the active site residues of AhR LBD (Fig. 5a-f).

**Table 1:** Binding energy and estimated binding constants of the AhR LBD-Ligand complexes calculated by Autodock

	Structure based binding pocket		Blind docking		3DLigandSite	
Ligand	Binding energy (kcal/mol)	Inhibition constant (μM)	Binding energy (kcal/mol)	Inhibition constant(μM)	Binding energy(kcal/mol)	Inhibition constant (μM)
TCDD	−5.53	87.98	**−7.17**	**5.53**	−6.07	35.38
FICZ	−5.63	75.21	**−8.18**	**1.01**	−6.49	17.56
I3C	−3.59	2350	−5.36	118.71	**−5.48**	**95.83**
DIM	−4.88	266.92	**−7.18**	**5.44**	−5.05	197.12
RES	−4.17	875.62	**−7.84**	**1.78**	−5.6	78.98
PTL	−5.73	63.08	**−7.29**	**4.51**	−6.04	37.14

The binding energy and inhibition constants for each AhRLBD-Ligand complex calculated at the residues predicted by each binding site prediction approach. The values shown in bold letters were considered for the MDS analysis

### MDS of AhR LBD and AhR LBD-ligand complexes

**Fig. 6:**
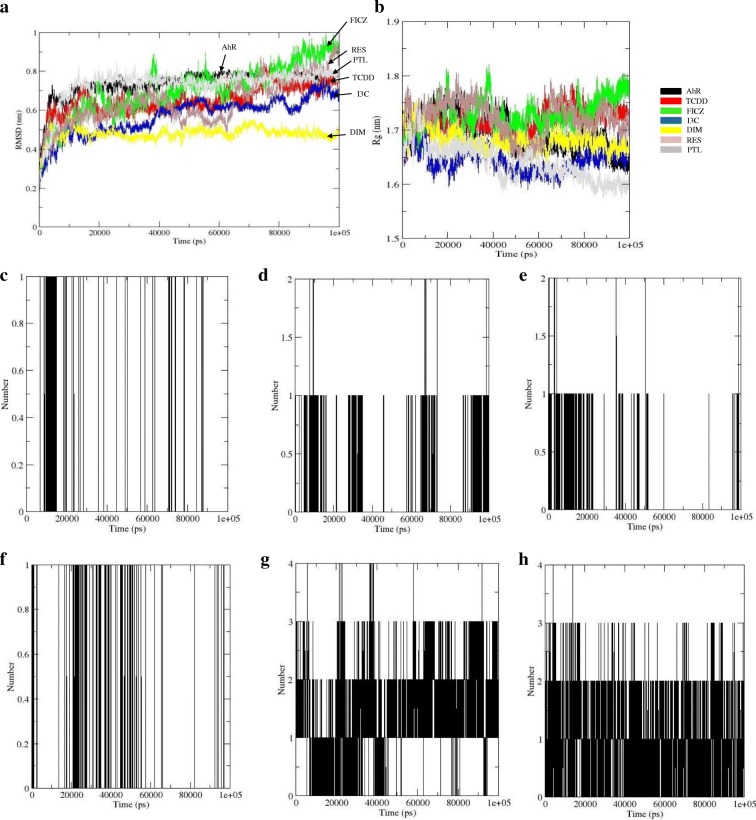
Conformational stability of the AhR LBD with each ligand during the 100 ns of MD simulations. **a** Represents the RMSD calculated for the AhR LBD and AhR LBD-ligand complexes. **b** Represents the Radius of gyration for the AhR LBD and AhR LBD ligand complexes. The average number of intermolecular Hydrogen bonds during 100 ns MDS for **c** AhRLBD-TCDD complex **d** AhRLBD-FICZ **e** AhRLBD-I3C **f** AhRLBD-DIM **g** AhRLBD-RES **h** AhRLBD-PTL. Black color lines represent the presence of H-bonds

To gain the conformational or functional insights into the mechanism of AhR LBD interactions with each ligand, we have performed MDS. To analyse and determine the stability and flexibility of binding for each ligand with AhRLBD, we have calculated the RMSD and radius of gyration (Rg) parameters during the simulation period. Results from backbone RMSD plotted as a function of time showed that both AhRLBD and AhRLBD-ligand complex structures exhibit deviations from their starting structure with a major fluctuation in the initial 10–40 ns followed by stable state after 50 ns for AhR LBD, with a stable fluctuation between 20 and 70 ns followed by a steady increase at the end of the simulation for AhRLBD-TCDD complex, with a stable fluctuation between 40 and 80 ns followed by a steady increase at the end of the simulation for AhRLBD-I3C complex, with a major fluctuation in the initial 10 ns followed by stable state after 20 ns for AhRLBD-DIM and AhRLBD-PTL complexes, whereas no stable state was observed for AhRLBD-FICZ and AhRLBD-RES complexes during the total 100 ns simulation time (Fig. 6a). A similar pattern was observed for Cα RMSD values (Additional file 5 A). Results from the average backbone RMSD values calculated after 100 ns in the AhRLBD and AhRLBD-ligand complex state showed a major difference in DIM (0.26 nm) and I3C (0.17 nm) bound complexes when compared to RES (0.09 nm), TCDD (0.08 nm), FICZ (0.03 nm) and PTL (0 nm) bound complexes (Additional file 6) with the AhRLBD. These results indicate that DIM and I3C bound complexes are less stable compared to the other ligand bound complexes. Further, FICZ showed the least difference in RMSD indicating that it is highly stable compared to the other ligands. Due to higher stability of interaction, FICZ has higher affinity towards AhRLBD compared to the other ligands. These results correlate with the hypothesis that due to stronger binding of FICZ to the AhRLBD active site it is able to activate AhR during T_H_17 cell development thereby markedly increasing the proportion of T_H_17 T cells, production of cytokines and exacerbated disease in experimental autoimmune encephalomyelitis [10]. The stability of the system was further evaluated by the radius of gyration (Rg) parameter which describes the overall compactness of protein. Results from backbone Rg showed a steady increase followed by a steady decrease after 20 ns for AhRLBD, a steady increase after 60 ns for AhRLBD-TCDD complex, a steady increase during the complete 100 ns simulation time for AhRLBD-FICZ, a steady decrease after 30 ns followed by a steady increase after 70 ns for AhRLBD-I3C, a steady decrease after 10 ns for AhRLBD-DIM, a steady decrease from 22 to 52 ns followed by a steady increase from 52 to 70 ns and a steady decrease from 70 to 100 ns for AhRLBD-RES, a steady decrease from 10 ns for AhRLBD-PTL (Fig. 6b). A similar pattern was observed in the Cα Rg plots (Additional file 5 B). Results from the comparison of average backbone Rg values with AhRLBD calculated over the 100 ns simulation showed much difference for PTL (0.06 nm) and I3C (0.05 nm) than FICZ (0.03 nm), RES (0.03 nm), TCDD (0.02 nm) and DIM (0.01 nm) bound complexes (Additional file 6).

**Fig. 7:**
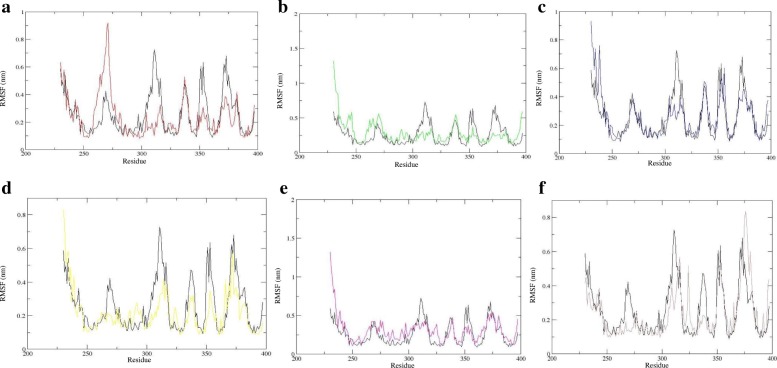
RMSF of AhR LBD and AhRLBD-ligand complexes during the MDS. RMSF plot of the backbone fluctuations calculated for **a** AhRLBD and AhRLBD-TCDD complex **b** AhRLBD and AhRLBD-FICZ complex **c** AhRLBD and AhRLBD-I3C complex **d** AhRLBD and AhRLBD-DIM complex **e** AhRLBD and AhRLBD-RES complex **f** AhRLBD and AhRLBD-PTL complex over 100 ns simulations. AhRLBD is shown in black color, AhRLBD-TCDD complex is shown in red color, AhRLBD-FICZ complex is shown in green color, AhRLBD-I3C complex is shown in blue color, AhRLBD-DIM complex is shown in yellow color, AhRLBD-RES complex is shown in magenta color, AhRLBD-PTL complex is shown in brown color

**Fig. 8:**
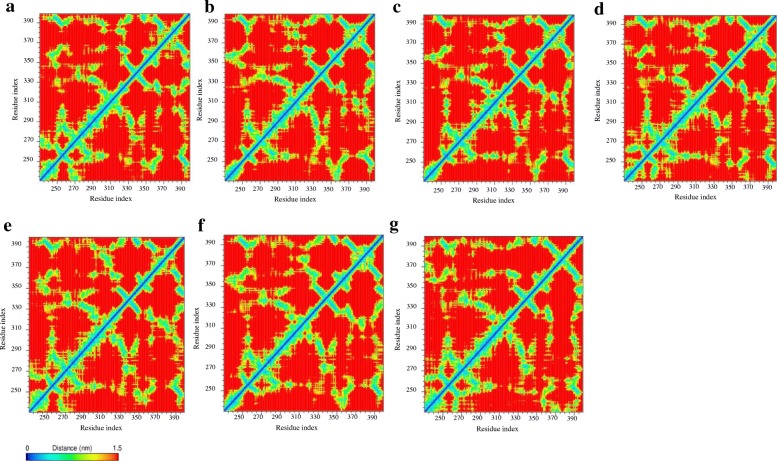
Different distance matrices depicting the smallest distance between residue pairs. The counter maps are calculated as the difference between the magnitude of pairwise distance fluctuations for the residues in **a** AhR LBD **b** AhRLBD-TCDD complex **c** AhRLBD-FICZ **d** AhRLBD-I3C complex **e** AhRLBD-DIM complex **f** AhRLBD-RES complex **g** AhRLBD-PTL complex during 100 ns MDS. The matrices are color coded, from red (higher distance) to blue (lower distance). The diagonal line represents the zero distances between the residues paired with themselves, while color spots represents the distances (nm) for each residue pair during 100 ns simulation

Results from the analysis of secondary structure elements during the simulation time showed a change in the AhRLBD-ligand complexes compared to AhRLBD (Additional file 5 c-i, Additional file 7). Results from the analysis of an average number of hydrogen bonds during the simulation time showed that in most of the AhRLBD-ligand complexes, docking level hydrogen bonding interactions shown by Protein-Ligand Interaction Profiler (PLIP) were maintained during the MDS (Fig. 6c-h). For AhRLBD-TCDD (Fig. 4c) and AhRLBD-DIM (Fig. 6f) complexes, the hydrogen bond occupancy between the AhRLBD protein amino acid residues and ligand atoms was lower compared to the AhRLBD-FICZ (Fig. 6d), AhRLBD-I3C (Fig. 6e), AhRLBD-RES (Fig. 6g) and AhRLBD-PTL (Fig. 6h) complexes. Results from the root mean square fluctuation (RMSF) during MDS showed a high flexibility in some of the residues for ligand bound complexes compared to the AhRLBD (Fig. 7a-f). Results from the distance matrices of the complexes showed a dissimilar pattern in mean smallest distance compared to the AhRLBD (Fig. 8a-h). Results from the distance matrices showed a conformational dynamics in the regions that has high fluctuations during the RMSD and RMSF analysis indicating that these regions show dynamic changes upon binding to these ligands. The blue and green colors in the plots indicate shorter distances between the residues (Fig. 8a-h).

### MM/PBSA binding free energy calculations for AhRLBD-ligand complexes

**Fig. 9:**
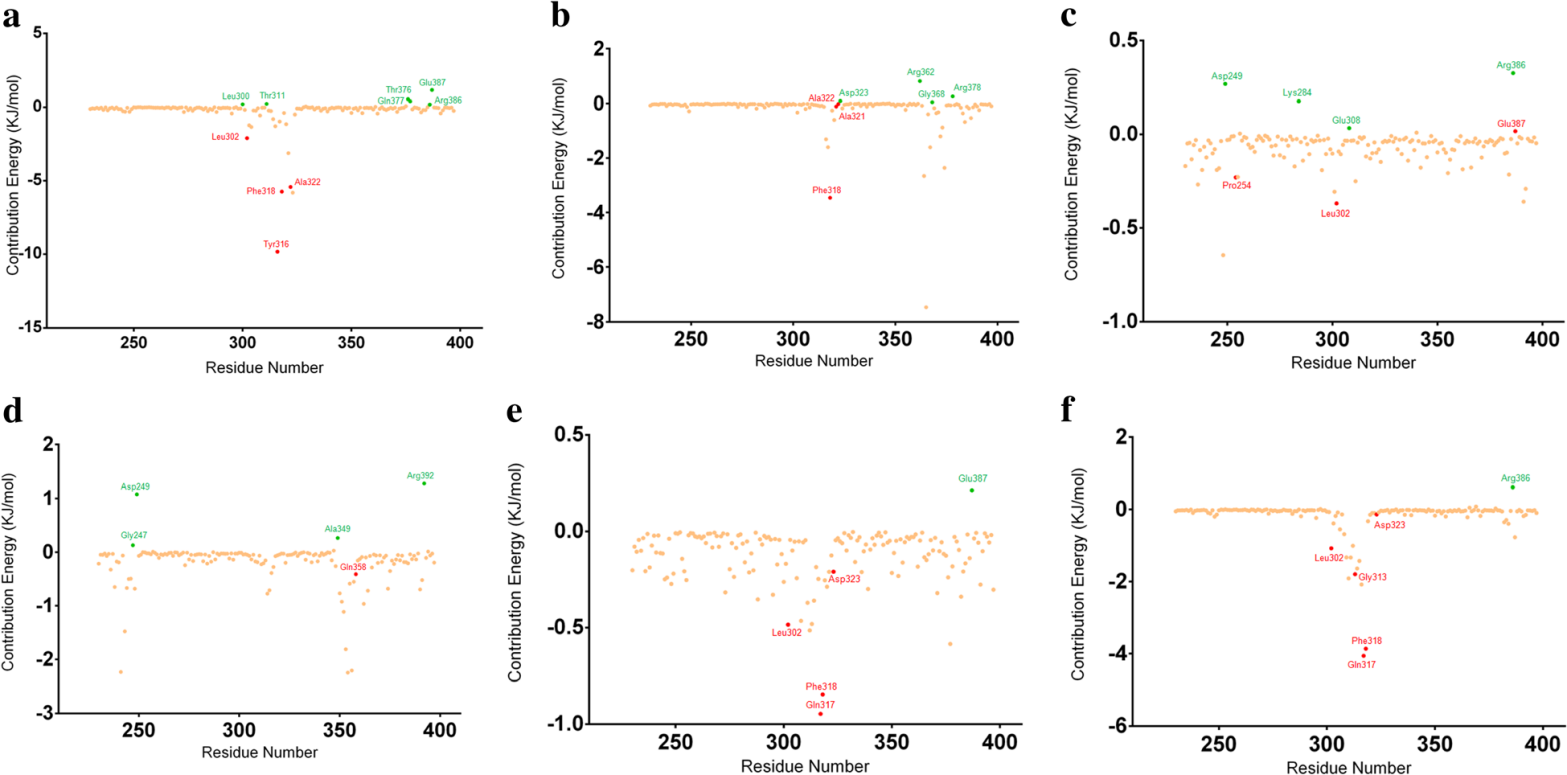
Per-residue decomposition analysis of AhR LBD in complex with each ligand. **a** Represents AhRLBD-TCDD complex **b** Represents AhRLBD-FICZ complex **c** Represents AhRLBD-I3C complex **d** Represents AhR LBD-DIM complex **e** Represents AhRLBD-RES complex **f** Represents AhRLBD-PTL complex. AhR LBD residues forming hydrogen boding interactions are shown in red color, residues with a positive contribution energy are shown in green color, other residues are shown in light orange color

To analyse the individual contributions of amino acid residues in AhRLBD to the interaction energies with the ligands, we have performed the binding free energy calculations. We estimated the favorable and unfavorable interactions based on estimated binding free energies. Results from the binding free energy calculations showed a different pattern of interaction energies for each of the AhRLBD bound ligand complexes (Additional file 7). Results from polar solvation energy, which is an unfavorable contribution to the binding free energy, appeared to be highly positive for FICZ, I3C, DIM, and PTL bound AhRLBD complexes (Additional file 7). Results for other energies such as vander Waals, electrostatic and non-polar solvation were found to be favorable for all the ligand bound complexes (Additional file 7). To characterize and identify the key residues of AhR LBD in each of the complexes, per-residue free energy decomposition analysis was performed so that we can elucidate their individual residue energy contributions, as shown in the Fig. 9a-f. Results from per-residue free energy decomposition analysis showed that some of the residues in AhR LBD are significantly contributing to binding with the ligands TCDD, FICZ, I3C, DIM, RES, and PTL.

## Discussion

AhR is a widely known transcription factor known to contribute to proper functioning of immune, hepatic, cardiovascular, vascular and reproductive systems [8], and its modulators have a potential role in the prevention/treatment of common human diseases/disorders [36]. Lack of experimentally determined structures for AhR has hampered any in-depth molecular understanding in providing the insight into the mechanisms of activation and transformation of the AhR. Thus, molecular modeling of the AhR structure and interactions can shed light on these ligand-dependent activation and transformation mechanisms [37]. Previously, several templates were proposed for generating AhR models based on the available Per-Arnt-Sim (PAS) structures at that time though they were not optimal [38]. Therefore, in the present study, we have used the recently resolved chain A crystal structure of mouse AhR PAS-A domain [39] as a template to generate the mouse AhR LBD model structure. Previous reports showed the binding pocket residues on AhR LBD for few known ligands [34, 35]. In the present study, we used three different computational methods to predict the binding residues on AhR LBD for the ligands TCDD, FICZ, I3C, DIM, RES, and PTL. These binding site residues were similar to the residues predicted using functional and site directed mutagensis experiments that were described previously [34, 35]. These AhR ligands were subjected to molecular docking and MDS at these predicted binding sites to analyze their respective mechanistic interactions with the AhRLBD.

MD trajectories are generally investigated as a specific marker to show the trends of energy and molecular deformations. Among the MDS parameters, RMSD is an important factor to analyse the equilibration of the trajectories thereby assessing the overall fluctuations. The difference in the average backbone and Cα RMSD values for AhRLBD-DIM and AhRLBD-I3C with the AhRLBD was high compared to other ligands indicating that these two ligands impose more fluctuations upon binding to AhR LBD and they are substantially distorted than the other ligands (Additional file 6). One of the important parameters to describe the equilibrium conformation of the total system is Rg [40]. According to the SCOPe classification [41], PAS domain belongs to the class d proteins (alpha and beta; α + β). Because we built our homology model using the AhR PAS-A domain; AhRLBD also has a fold similar to class d proteins. Results from the average backbone and Cα Rg values for AhRLBD showed 1.69 ± 0.0 nm (16.9 Å) which is in accordance with the previous results where SCOP class d proteins with a 151–200 residue size show a Rg value of 16.9 ± 0.1 Å [40]. The average backbone Rg values for I3C and DIM bound AhRLBD complexes (Additional file 6) was higher than other bound ligands indicating a global conformational change in AhRLBD during the simulation upon binding to these two ligands. These results are in agreement with the previous reports that agonist ligands induce a conformational change in the mouse AhR [42].

To understand the secondary structural profile changes in more detail, during the simulation, we have carried out the analysis for both the AhRLBD and AhRLBD-ligand complexes using DSSP. A major structural change occurred in the α-helical regions with residues found distorted during 60–85 ns for AhRLBD-TCDD complex (Additional file 5 D), α-helical residues and turn residues for AhRLBD-FICZ complex (Additional file 5 E), α-helices and β-sheets for AhRLBD-I3C complex (Additional file 5 F), coils for AhRLBD-DIM complex (Additional file 5 G), α-helices and β-sheets for AhRLBD-RES complex (Additional file 5 H) and α-helices, β-sheets and coils for AhRLBD-PTL complex (Additional file 5 I) in comparison to AhRLBD (Additional file 5 C and Additional file 8).

In general, the H-bond interactions during the docking simulations provide a static map of the interactions. To analyse whether these contacts were maintained in the AhRLBD and AhRLBD-ligand complexes, we mapped the H-bond interactions during the simulation time (Fig. 6c-h). For AhRLBD-TCDD and AhRLBD-DIM complexes, the H-bonds formed by Phe318, Ala322 (Fig. 6c) and Gln358 (Fig. 6f) were stable throughout the MDS. For AhRLBD-FICZ complex, the H-bonds formed by Phe318, Alal321, and Ala322 were stable throughout the simulation with slight fluctuations at 10.5 and 67 ns of the MDS (Fig. 6d). In AhRLBD-I3Ccomplex, H-bonds formed by the residues Pro254, Leu302 and Glu387 showed slight fluctuations at 3, 44, 50 and 97 ns during the MDS (Fig. 6e). Because PTL is an analog of RES, both of these ligands showed almost a similar pattern of H-bond occupancy with a slight fluctuation during the simulation time (Fig. 6g and h). Overall, these results showed that the H-bond interactions formed by the ligands TCDD and DIM with the AhRLBD residues were comparatively stable than the other ligands. To quantify the flexibility at individual residue positions during our MDS, we have calculated the root mean square fluctuation (RMSF) of the backbone atoms of each residue for AhRLBD and AhRLBD-ligand complexes (Fig. 7a-f). A higher fluctuation was observed in the extended beta sheet 2nd (257–261) and 3rd (282–283), coil 1st and turn 3rd (262–275), helix 2nd (276–279), turn 4th (280–281) for AhRLBD-TCDD complex (Fig. 7a). Upon evaluation of RMSF, the difference in the RMSF of binding site residue Ala322 was found to be the largest between the TCDD bound and unbound states of AhRLBD indicating that the binding of TCDD affected the dynamics of this residue. In AhRLBD-FICZ complex, higher fluctuations were observed in the helix 1st (230–240), 2nd (276–279), 3rd ^(^285–287) and 4th (290–295), turn 1st (241–244), 2nd (254–256), 3rd (262–275) and 4th (280–281), extended beta sheet 1st (247–253), 2nd (257–261) and 3rd (282–283), coil 2nd (289), beta bridge 1st (288) (Fig. 7b). Evaluation of RMSF difference for binding site residue for FICZ bound and unbound states of AhRLBD showed higher fluctuations in the residues Ala321 and Ala322 indicating that these two residues showed change in stability upon FICZ binding to the AhRLBD. In AhRLBD-I3C and AhRLBD-DIM complexes, higher fluctuations were observed in the helix 1 (230–240) and turn 1 (241–242) residues of AhRLBD (Fig. 7c and d). Evaluation of RMSF difference for binding site residues for I3C and DIM bound and unbound states of AhRLBD showed higher fluctuations in the residue Leu302 for AhRLBD-I3C complex whereas no significant higher fluctuations were observed for the binding residues in AhRLBD-DIM complex. These results showed that the residue Leu302 is functionally important for binding AhRLBD to I3C.

In AhRLBD-RES complex, higher fluctuations were observed in the helix 1st (230–240), 2nd (276–279) and 3rd (284–287) and 4th (290–300), turn 1st (241–243), 2nd (254–256) and 3rd (262–268, 273–275), 5th (301–304), 6th (322–325) and 11th (379–384), extended beta sheet 1st (253), 2nd (257–261) and 4th (328), 6th (361–366) and 11th(385–395), beta bridge 1st (288), 2nd (326) and 5th (361), coils 2nd (289), 6th (327) and 11th (396–397) (Fig. 7e). RMSF difference for binding site residue for RES bound and unbound states of AhRLBD showed higher fluctuations in the residues Leu302, Phe318, and Asp323 indicating that binding of RES affected the dynamics of these residues. In AhRLBD-PTL complex, higher fluctuations were observed in the extended beta sheet 4th (328–39), 6th (362–366) and 11th (385–395), beta bridge 2nd (326), 4th (360) and 5th (361), coils 5th (319–325), 6th (327), 10th (374–381) and 11th (396–397) (Fig. 7f). RMSF difference for binding site residue for PTL bound and unbound states of AhRLBD showed higher fluctuations in the residues Gln317 and Asp323 indicating that PTL induce flexibility among these residues upon binding to AhRLBD. The average RMSF of the residues in the AhR ligand bound and unbound complexes were as follows: AhRLBD-RES (0.30 nm) > AhRLBD-FICZ (0.28 nm) > AhRLBD-I3C (0.26 nm) > AhR (0.25 nm) > AhRLBD-TCDD (0.23 nm) ~ AhRLBD-PTL (0.23 nm) > AhRLBD-DIM (0.21 nm).

The distance matrix is a widely used structural analysis approach to capture collective domain motions in addition to clearly visualizing the conformational change between two states of a protein [43]. Here we used the same approach to visualize the collective domain motions along with conformational changes for ligand bound and unbound states of AhRLBD. Results showed that the conformation strain of the residues differs prominently between ligand bound and unbound states of AhRLBD. The comparison between maps with or without ligands allowed us to estimate the growing gap between each block following the interaction of AhRLBD with each of the ligands (Fig. 8a-g). We then investigated the binding residues of the AhRLBD showing the interaction with each ligand in the context of these contact maps (Additional file 9 A-F). The conformation of these residues showed a prominent difference for each of these ligands bound complexes. Hydrophobic residues Leu302, Tyr316, Phe318, Ala322 interacting with TCDD in AhRLBD-TCDD complex showed a low value indicating a minimum escalation in the flexibility of the conformation in the AhRLBD upon binding to TCDD (Additional file 9 A). The same pattern was observed for the hydrophobic residues Phe318, Ala321, Ala322 in AhRLBD-FICZ complex (Additional file 9 B) and polar residue Gln358 in AhRLBD-DIM complex (Additional file 9 D). These results indicated that the ligands TCDD, FICZ and DIM interactions minimize the distance between these AhRLBD binding residues thereby minimizing the overall flexibility by changing its closed conformation to open form. A different pattern was observed for the complexes formed by I3C, RES, and PTL where the hydrophobic residues Leu302, Phe318 showed minimum distances whereas other residues showed higher values of distances between the residue pairs (Additional file 9 C, E and F).

To identify important binding site residues and characterize how interactions may change as a result of each ligand, structural and energetic molecular “footprints” were computed for each MD trajectory through MMPBSA binding free energy calculations. Each of these footprints represent the per-residue decomposition of interactions, averaged over the production simulations, between each AhR LBD residue and the ligand. Because the g_mmpbsa tool has certain limitations in providing the binding free energies, we only considered ΔE_vdW_ (Van der Waal) and ΔG_SASA_ (Non-Polar solvation energy calculated based on SASA) energies for our analysis. Results showed that these calculations (Additional file 7) were in agreement with the molecular docking and MDS results. Further, to determine the energy contributions of the key AhR LBD residues interacting with each ligand, a per-residue decomposition analysis was performed. In AhRLBD-TCDD complex, residues Leu300, Thr311, Thr376, Gln377, Glu387, Arg386 disfavoured binding whereas hydrogen bonding residues Leu302, Tyr316, Phe318 and Ala322 shown by PLIP (Fig. 9a) favoured binding which is in agreement with the results from RMSF analysis with Ala322 showing large fluctuations, thereby confirming that Ala322 is a key residue in AhRBD binding to TCDD. In AhRLBD-FICZ complex, residues Asp323, Arg362, Gly368 and Arg378 disfavoured binding whereas hydrogen bonding residues Phe318, Ala321 and Ala322 shown by PLIP (Fig. 9b) favoured binding which is in agreement with the results from RMSF analysis with Ala321 and Ala322 showing higher fluctuations thereby confirming that these two residues Ala321 and Ala322 are key in AhR LBD binding to FICZ. In AhRLBD-I3C complex, residues Asp249, Lys284, Glu308, Arg386 disfavoured binding whereas among the hydrogen bonding residues shown by PLIP, Glu387 disfavoured binding whereas Pro254 and Leu302 (Fig. 9c) favoured binding which agrees with the results from RMSF analysis with Leu302 showing higher fluctuations thereby confirming that the residue Leu302 is key in AhR LBD binding to I3C. In AhRLBD-DIM complex, residues Gly247, Asp249, Ala349, Arg392 disfavoured binding whereas hydrogen bonding residue Gln358 shown by PLIP favoured binding (Fig. 9d). In AhRLBD-RES complex, residues Glu387 disfavoured binding whereas hydrogen bonding residues Leu302, Gln317, Phe318 and Asp323 shown by PLIP (Fig. 9e) favoured binding which is in agreement with the results from RMSF analysis with Leu302, Phe318 and Asp323 showing higher fluctuations thereby confirming that these three residues Leu302, Phe318 and Asp323 are key in AhR LBD binding to RES. In AhRLBD-PTL complex, residues Arg386 disfavoured binding whereas hydrogen bonding residues Leu302, Gly313, Gln317, Phe318 and Asp323 (Fig. 9e) shown by PLIP favoured binding, which agrees with the results from RMSF analysis with Leu302, Phe318 and Asp323 showing higher fluctuations thereby confirming that these three residues Leu302, Phe318 and Asp323 were key in AhR LBD binding to RES.

## Conclusions

We have performed molecular modeling, molecular docking, competitive binding assay followed by molecular dynamic simulations to evaluate the interactions of selected AhR ligands towards AhRLBD. Our study provided insights about the interaction details of each AhR ligand with the AhRLBD. Some of these ligands showed some flexibility inside the binding site allowing them to adopt a favourable conformation as observed through MMPBSA results. AhR being a novel receptor for various pathways and diseases, results from the calculations performed in our study will provide a valuable benchmark for the researchers working in this area.

## Methods

### Molecule preparation

The structure of the AhR ligands, TCDD (Compound ID: 15625), FICZ (Compound ID: 1863), I3C (Compound ID: 3712), DIM (Compound ID: 9856273), RES (Compound ID: 445154) and RES analog PTL (Compound ID: 667639) were downloaded from the PubChem compound database [44]. Chemical structures of each ligand are provided in the Fig. 1.

### Sequence retrieval, homology modelling and validation

The homology model for the mouse AhR ligand binding domain (AhRLBD) was constructed (Fig. 2a) using Modeller version 9.14 [45]. The amino acid sequence of the ligand binding domain of mouse AhR (entry ID: P30561) [46, 47] was retrieved from the UniProt database [48]. The template search for AhR LBD was performed using NCBI BLAST search against Protein Data Bank (PDB) [49]. The structure model was built using the recently solved chain A crystal structure of mouse AhR PAS-A domain (PDB ID: 4M4X) [32] with a sequence identity percentage greater than 30% instead of previously proposed templates [37, 39, 50]. The modelled structure was refined using Modrefiner [51], an algorithm which generates the refined full-atom models from Cα traces with improved global and local qualities. Its refinement procedure involves the construction of a main-chain model from the Cα trace with acceptable backbone topology and main-chain hydrogen (H)-bonding network followed by the addition of side-chain atoms onto the backbone conformation and optimization using a composite physics and knowledge-based force field. The refined model was subjected to energy minimization using the Gromacs 5.0.4 package [52]. Finally, the generated model (Fig. 2a) was validated for quality using the ProSA, a web based server that is widely used to check 3D models of protein structures for potential errors [53] and Ramachandran plot available at the Rampage server [54]. Details of the sequence to template structure alignment generated using Align2D module of modeller version 9.14. and the secondary structure analysis for the modelled mouse AhR LBD structure was provided (Additional file 2 A, B).

### Binding site prediction

In general, recognition of the binding site residues is vital for elucidating the function of a protein. Experimentally predicting these binding site residues is often expensive and time consuming. Therefore, computational prediction methods are very handy in these situations. These computational methods are primarily classified into sequence-based methods, structure-based methods and hybrid methods [55]. Each of these methods has its own disadvantages. To improve the accuracy of binding site prediction, we used three approaches for our study: i) Structure-based alignment method: Initially, we identified the homologous structures with bound ligands using the 3D BLAST search against the nr-PDB ID [56]. Predicted homology structures were superimposed using the Mulitprot [57] and Mustang [58] servers. The ligands in the homology structures were superimposed onto the protein structure to predict the ligand binding site. ii) Blind docking approach: Previously, several studies showed that blind docking is an effective and novel approach in a situation where the binding site for a ligand is unknown [59, 60]. In the present study, we used the same approach to identify the potential binding sites for each ligand on the AhRLBD using Autodock 4.2.6 [61]. iii) We used 3DLigandSite, a web server which predicts ligand-binding sites with Matthew’s correlation coefficient of 0.64 [62].

### Molecular docking

Molecular docking has been used as a successful tool to explain the mechanism in several reports showed previously [63–65]. Therefore, to analyse the mechanism of interaction of these ligands with AhR LBD, we have performed molecular docking at the predicted binding sites using the program Autodock 4.2.6. The input files for the molecular docking was generated using pyrx program [66]. For molecular docking the AhR ligands with AhRLBD, we used empirical free energy function and Lamarckian genetic algorithm (LGA) with the following settings: a maximum number of 2,500,000 energy evaluations, an initial population of 150 randomly placed individuals, a maximum number of 27,000 generations, a mutation rate of 0.02, a crossover rate of 0.8, and an elitism value (number of top individuals to survive to next generation) of 1. We applied the Solis and Wets algorithm with a maximum of 300 iterations per search for local search. For all the unmentioned parameters, we considered the default values. The generated best poses of the docking run for AhRLBD and each AhRLBD-ligand complex, was have evaluated according to the binding energy and estimated inhibition constant scoring function implemented in Autodock. The interactions between AhRLBD residues and the respective ligands were visualized using protein-ligand interaction profiler(PLIP) [67].

### Competitive binding of AhR ligands with mouse AhR

Previously, several studies have been successful in elucidating the protein ligand interactions by performing molecular docking experiments followed by competitive binding studies [68, 69]. To investigate the interactions and binding efficiency of the mouse AhR with its ligands, we have performed the competitive binding assay experiments in vitro based on the method developed by Gasiewicz and Neal [70]. C57BL/6 mice were obtained from Jackson Laboratories (Bar Harbor, ME) and housed in an AAALAC accredited animal facility in the University of South Carolina. At age 12–14 weeks, mice were euthanized by overdose of isoflurane inhalation, a method approved by the Panel on Euthanasia of the American Veterinary Medical Association and recommended by local IACUC (institutional animal care and use committee). Livers from mice were homogenized in HEDG buffer (25 mM HEPES pH 7.4, 1.5 mM EDTA, 10% glycerol) and centrifuged at 10,000 g for 30 min. The supernatant was centrifuged again at 105,000 g for 60 min. The cytosol was collected and diluted with HEDG buffer to the protein concentration of 2 mg/ml. A concentration of 3 nM ^3^H-TCDD (ARC, St. Louis, MO) and various concentrations of a competitive AhR binding ligand was added to 0.2 ml of liver cytosol and the mixture was incubated at 20^▫^C for 2 h. HTP (hydroxyapatite, Bio-Rad) (0.2 ml) suspended in HEDG buffer was added to the reaction mixture and incubated at 4 °C for 30 min with rotation. HTP was pelleted by centrifugation and washed with HEDG buffer containing 0.5% Tween 80 for 3 times. After the last wash, 1 ml of ethanol was added to the HTP pellet. The radiation counts in ethanol were measured by liquid scintillation counting. The relative binding affinity was determined by calculating the percentage of cytosolic bound ^3^H-TCDD in the presence of a competitor to that in the absence of a competitor.

### Molecular dynamic simulations (MDS)

MDS delivers dynamical structural information of biomacromolecules and a treasure of active information about the protein and ligand interactions, which is very significant in understanding the core of interactions [71]. To analyse the dynamical structural information of AhRLBD and AhRLBD-ligand complexes we have performed MDS using the Gromacs 5.0.4 package at a 100 nano seconds (ns) time scale [52]. The evaluated pose of the docking run for AhRLBD and AhRLBD-ligand complexes according to the binding energy and estimated inhibition constant scoring function implemented in Autodock were used as a starting point for all-atom MDS in explicit water. To describe the system’s topology for the protein and protein-ligand complexes, we chose the OPLS-AA/L all-atom force field [72] which has been used as a force field to study MDS in AhR previously [73] and solvated with tip3p [74, 75] water molecules. The neutral charge of the system was maintained by adding the Na^+^ and Cl^−^ counter ions. Simulations were performed in the NPT and NVT ensemble, using the Parrinello barostat [76] with a time constant τ = 2 ps and the V-rescale thermostat [77] with a time constant τ = 0.1 ps and a time step dt = 2 fs. For the electrostatic and van der Waals interactions, we employed the Partial Mesh Ewald (PME) algorithm [78]. All bond lengths were constrained using the LINCS algorithm [79]. Energy minimization of the system was performed using the steepest descent algorithm with a maximum step size of 0.01 nm. The system was subjected to equilibration at a 300 K temperature and 1 bar pressure. Finally, we performed seven simulations (AhRLBD, AhRLBD-TCDD, AhRLBD-FICZ, AhRLBD-I3C, AhRLBD-DIM, AhRLBD-RES, AhRLBD-PTL) with 100 ns each and the atom coordinates were recorded every 2 ps during the simulation for later analyses.

### Analysis of MDS trajectories

Comparative analysis of structural deviations in the protein (AhRLBD) and protein-ligand complexes (AhRLBD-TCDD, AhRLBD-FICZ, AhRLBD-I3C, AhRLBD-DIM, AhRLBD-RES, AhRLBD-PTL) such as root-mean-square deviation (RMSD), root-mean-square fluctuation (RMSF), solvent-accessible surface area (SASA), secondary structure calculation etc., were computed using g_rms, g_rmsf, g_sas and g_gyrate built-in functions of GROMACS package. Presence of hydrogen bonds during the simulations was evaluated using the g_h bond tool in GROMACS with default cut-off angle value of 30° and a cut-off radius of 0.35 nm.

### Contact map calculations

To calculate the contact map for residues in AhR ligand bound and unbound states, we used g_mdmat in Gromacs, which predicts the distance matrices consisting of the smallest distances between residue pairs. Frames during the 100 ns time scale MDS was used for the calculation of contact maps.

### MM-PBSA approach-based interaction energy estimation

MM/PBSA and MM/GBSA calculations have been applied to a large number of systems successfully reproducing, rationalizing the experimental findings and improving the results of virtual screening and docking [80]. The MM-PBSA calculations for each AhRLBD-ligand complex was determined using the g_mmpbsa tool [81]. For each simulated system, from the last 20 ns of the MD trajectory, we have considered 2000 snapshots of the complexes with a 10 ps intervals spacing to ensure a low statistical error thereby ensuring that the structures are uncorrelated [82]. The binding free energy of each complex for every snapshot is calculated using the following set of equations as described previously [83–85]:

1 $$ {\Delta \mathrm{G}}_{\mathrm{bind}}={\mathrm{G}}_{\mathrm{complex}}\hbox{-} \left({\mathrm{G}}_{\mathrm{protien}}+{\mathrm{G}}_{\mathrm{ligand}}\right) $$

2 $$ {\Delta \mathrm{G}}_{\mathrm{bind}}={\Delta \mathrm{E}}_{\mathrm{MM}}+{\Delta \mathrm{G}}_{\mathrm{sol}}\hbox{-} \mathrm{T}\Delta \mathrm{S} $$

3 $$ {\Delta \mathrm{E}}_{\mathrm{MM}}={\Delta \mathrm{E}}_{\mathrm{elec}}+{\Delta \mathrm{E}}_{\mathrm{vdW}} $$

4 $$ {\Delta \mathrm{G}}_{\mathrm{sol}}={\Delta \mathrm{G}}_{\mathrm{pol}}+{\Delta \mathrm{G}}_{\mathrm{npol}} $$

5 $$ {\Delta \mathrm{G}}_{\mathrm{npol}}={\upgamma^{\ast}}_{\mathrm{SASA}}+\mathrm{b} $$

where ΔG_bind_ is the total binding free energy, G_complex_, G_protein_ and G_ligand_ are the energies for the AhRLBD-ligand complexes, protein (AhRLBD) and the ligands (TCDD, FICZ, I3C, DIM, RES, PTL) respectively. The binding energy can also be denoted as the eqs. 2 and 3 where ΔE_elec_ is the electrostatic interaction energy and ΔE_vdW_ is the vander Waals interaction energy. The solvation energy (ΔG_sol_) is decomposed into polar (ΔG_pol_) and nonpolar solvation energy (ΔG_npol_) components. ΔG_npol_ was calculated using the eq. 5 where γ is a coefficient related to surface tension of the solvent, SASA is the solvent accessible surface area and b is the fitting parameter. Polar solvation energies were calculated using linear Poisson-Boltzmann equation whereas non-polar solvation energies were calculated with the solvent accessible surface area with an offset value (b) of 3.84928 kJ.mol^− 1^ and surface tension proportionality (γ) set at 0.0226778 kJ.mol^− 1^.Å^− 2^. Contribution of individual protein residues to the three energetic components was determined through per-residue decomposition.

### Statistics and graphical analysis

To calculate the contact map for residues in AhR ligand bound and unbound states, we used g_mdmat in Gromacs, which predicts the distance matrices consisting of the smallest distances between residue pairs. Frames during the 100 ns time scale MDS was used for the calculation of contact maps. All statistical analyses were performed using GraphPad Prism 6.0 for windows (GraphPad Software, San Diego, CA). Graphs obtained from MDS were plotted using GRACE software (http://plasma-gate.weizmann.ac.il/Grace/). Molecular visualization of the proteins was performed using UCSF Chimera [86].

### Computing specifications

All MDS used in the study were performed using Research Cyber infrastructure, University of South Carolina and Comet XSEDE cluster at Xsede High-Performance computing resource portal. Calculations such as docking studies, ensemble calculations, trajectory analysis and other calculations were performed on local computer.

## Additional files

Additional file 1: 3D BLAST search for structure homologs of mouse AhR LBD. The following PDB structures were predicted using 3D BLAST search. (DOCX 15 kb)

Additional file 2: Alignment, secondary structure and Lesk-Hubbard plot analysis. **A** Alignment of mouse AhR LBD with the template (Chain A of the 4M4X) using Align2D module of modeller version 9.14. * at the bottom of the alignment represent the conserved residues between the two sequences. **B** Represents the assignment of AhR LBD residues to secondary structure elements using the STRIDE server. **C** Lesk-Hubbard plot showing the Root-Mean-Square Deviation (RMSD)-based molecular sieving and the number of residue correspondences performed using the MUSTANG server. (TIF 259 kb)

Additional file 3: Interactions of ligands with AhR LBD. **A-F** Represent the AhR LBD interactions with ligands using Structure based binding pocket approach **G-K** Represent the AhRLBD interactions with ligands using Ligsite server **L** Represent the AhRLBD interactions with ligands using blind docking approach. TCDD is shown in salmon red color sticks, FICZ is shown in cyan color sticks, I3C is shown in light orange color sticks, DIM is shown in green color sticks, RES is shown in purple color sticks, PTL is shown in limon color sticks. AhR LBD residues is shown in sky blue color sticks. Hydrogen bonding interactions were shown in yellow color and hydrophobic interactions were shown in grey color lines. (TIF 468 kb)

Additional file 4: Summary of interacting amino acid residues with the various AhR ligands under study upon docking in each of the predicted binding site. Residues forming H-bonds are shown in bolded italics. (DOCX 14 kb)

Additional file 5: Conformational changes. **A** Represent the Cα RMSD values of the AhRLBD and its ligands **B** Represents the Cα Rg of the AhRLBD and its ligands. Time evolution of Secondary structure elements during the 100 ns MDS for **C** AhR LBD **D** AhRLBD-TCDD complex **E** AhRLBD-FICZ **F** AhRLBD-I3C **G** AhRLBD-DIM **H** AhRLBD-RES **I** AhRLBD-PTL. The color scale at the bottom of each plot represents the secondary structure elements classified based on DSSP classification of each secondary structure element. (TIF 1805 kb)

Additional file 6: Average Summary of interacting amino acid residues with the various AhR ligands under study upon docking in each of the predicted binding site. (DOCX 13 kb)

Additional file 7: Calculated binding energies using MD-MM/PBSA or direct MM/PBSA for the six AhR-ligand complexes. (DOCX 13 kb)

Additional file 8: Percentage of secondary structure elements during the 100 ns simulation. The percentages were calculated using the DSSP program in Gromacs. (DOCX 14 kb)

Additional file 9: Mean smallest distance for the interacting residues. **A** Represents the interacting residues in AhRLBD and AhRLBD-TCDD complex **B** Represents the interacting residues in AhRLBD and AhRLBD-FICZ complex **C** Represents the interacting residues in AhRLBD and AhRLBD-I3C complex **D** Represents the interacting residues in AhRLBD and AhRLBD-DIM complex **E** Represents the interacting residues in AhRLBD and AhRLBD-RES complex **F** Represents the interacting residues in AhRLBD and AhRLBD-PTL complex. Residues in AhRLBD shown in grey color; AhRLBD-TCDD complex residues shown in red color; AhRLBD-FICZ complex residues shown in cyan color; AhRLBD-I3C complex residues shown in orange color; AhRLBD-DIM complex residues shown in green color; AhRLBD-RES complex residues shown in violet color; AhRLBD-PTL complex residues shown in pale yellow color. (TIF 119 kb)

## Abbreviations

AhR: Aryl hydrocarbon receptor
AhRLBD: AhR ligand binding domain
DIM: 3,3′-diindolylmethane
FICZ: 6-formylindolo[3,2-b]carbazole
I3C: Indole-3-carbinol
ITE: 2-(1′H-indole-3′-carbonyl)-thiazole-4-carboxylic acid methyl ester
LGA: Lamarckian genetic algorithm
MDS: Molecular dynamic simulation
PLIP: Protein-Ligand Interaction Profiler
PTL: Piceatannol
RES: Resveratrol
Rg: Radius of gyration
RMSD: Root-mean-square deviation
RMSF: Root mean square fluctuation
SASA: Solvent-accessible surface area
TCDD: 2,3,7,8-tetrachlorodibenzo-ρ-dioxin
Th: T helper Tregs: T regulatory cells
ΔE_elec_: Electrostatic interaction energy
ΔE_vdW_: Vander Waals interaction energy
ΔG_npol_: Nonpolar solvation energy (ΔG_npol_) components
ΔG_pol_: Polar energy
ΔG_sol_: Solvation energy
