# Supplementary material for: Comparative analysis of interactions between aryl hydrocarbon receptor ligand binding domain with its ligands: a computational study
Source: BMC Struct Biol. 2018 Dec 6;18:15. doi: 10.1186/s12900-018-0095-2 (PMC6282305; doi:10.1186/s12900-018-0095-2)
Supplement: Supplementary file 1 — 3D BLAST search for structure homologs of mouse AhR LBD. The following PDB structures were predicted using 3D BLAST search. (DOCX 15 kb) [file 12900_2018_95_MOESM1_ESM.docx]

**Additional file 1. 3D BLAST search for structure homologs of mouse AhR LBD.** The following PDB structures were predicted using 3D BLAST search

| **PDB ID** | **Chain** | **Length** | **Score** | **E-value** | **%Identity** | **%Gaps** |
| --- | --- | --- | --- | --- | --- | --- |
| 3f1n | A | 53 | 66.9 | 2e-12 | 47.2 | 5.7 |
| 2vlg | A | 55 | 63.5 | 2e-11 | 45.5 | 7.3 |
| 1s66 | L | 68 | 60.5 | 2e-10 | 35.3 | 11.8 |
| 1wa9 | A | 59 | 56.7 | 2e-09 | 35.6 | 5.1 |
| 3cax | A | 131 | 55.9 | 4e-09 | 32.1 | 13.7 |
| 3gdi | A | 63 | 54.4 | 1e-08 | 38.1 | 6.3 |
| 3lyx | A | 65 | 54.0 | 2e-08 | 29.2 | 4.6 |
| 3f1n | B | 54 | 52.8 | 3e-08 | 33.3 | 5.6 |
| 3kxr | A | 81 | 50.2 | 2e-07 | 34.6 | 13.6 |
| 3mxq | A | 91 | 50.2 | 2e-07 | 29.7 | 13.2 |
| 1v9y | A | 57 | 50.2 | 2e-07 | 33.3 | 5.3 |
| 3gec | A | 63 | 49.0 | 5e-07 | 33.3 | 14.3 |
| 2yzq | A | 84 | 48.3 | 8e-07 | 31.0 | 17.9 |
| 1r4y | A | 83 | 47.9 | 1e-06 | 34.9 | 16.9 |
| 3a0s | A | 51 | 47.9 | 1e-06 | 43.1 | 7.8 |
| 3no3 | A | 80 | 47.1 | 2e-06 | 36.2 | 15.0 |
| 3mfx | A | 59 | 46.8 | 2e-06 | 39.0 | 11.9 |
| 1zcc | A | 62 | 46.8 | 2e-06 | 35.5 | 16.1 |
| 1f98 | A | 103 | 46.8 | 2e-06 | 31.1 | 14.6 |
| 3ewk | A | 108 | 46.4 | 3e-06 | 29.6 | 14.8 |
