# Supplementary figures and images for: Comparative analysis of interactions between aryl hydrocarbon receptor ligand binding domain with its ligands: a computational study
Source: BMC Struct Biol. 2018 Dec 6;18:15. doi: 10.1186/s12900-018-0095-2 (PMC6282305; doi:10.1186/s12900-018-0095-2)

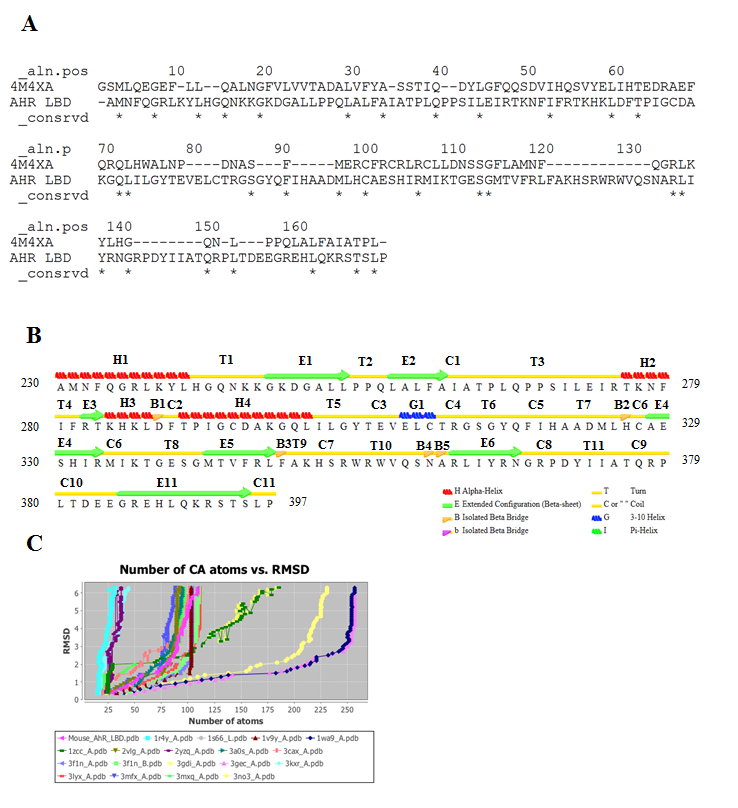

Supplement: Supplementary file 2 — Alignment, secondary structure and Lesk-Hubbard plot analysis. A Alignment of mouse AhR LBD with the template (Chain A of the 4M4X) using Align2D module of modeller version 9.14. * at the bottom of the alignment represent the conserved residues between the two sequences. B Represents the assignment of AhR LBD residues to secondary structure elements using the STRIDE server. C Lesk-Hubbard plot showing the Root-Mean-Square Deviation (RMSD)-based molecular sieving and the number of residue correspondences performed using the MUSTANG server. (TIF 259 kb) [file 12900_2018_95_MOESM2_ESM.tif]

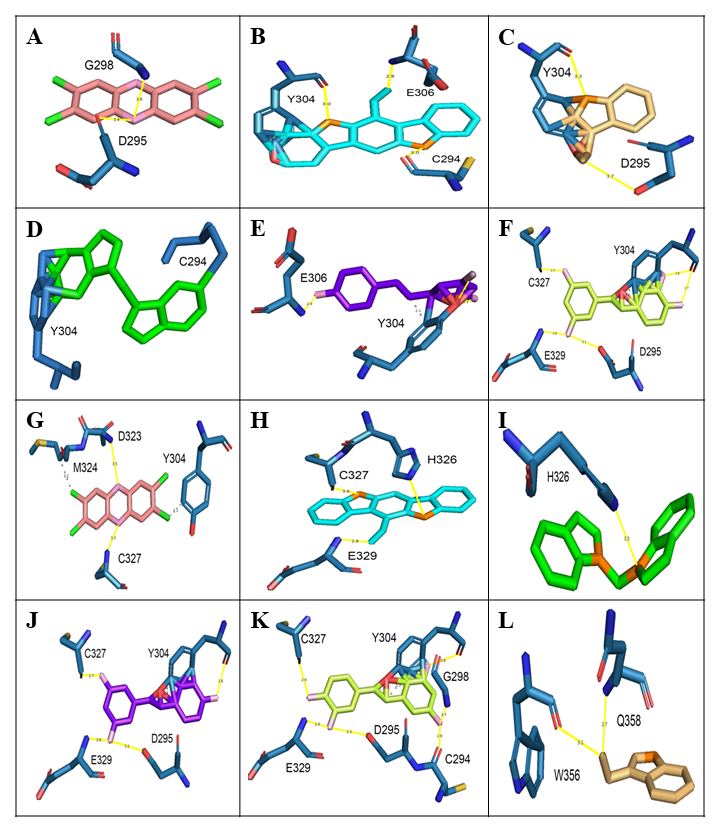

Supplement: Supplementary file 3 — Interactions of ligands with AhR LBD. A-F Represent the AhR LBD interactions with ligands using Structure based binding pocket approach G-K Represent the AhRLBD interactions with ligands using Ligsite server L Represent the AhRLBD interactions with ligands using blind docking approach. TCDD is shown in salmon red color sticks, FICZ is shown in cyan color sticks, I3C is shown in light orange color sticks, DIM is shown in green color sticks, RES is shown in purple color sticks, PTL is shown in limon color sticks. AhR LBD residues is shown in sky blue color sticks. Hydrogen bonding interactions were shown in yellow color and hydrophobic interactions were shown in grey color lines. (TIF 468 kb) [file 12900_2018_95_MOESM3_ESM.tif]

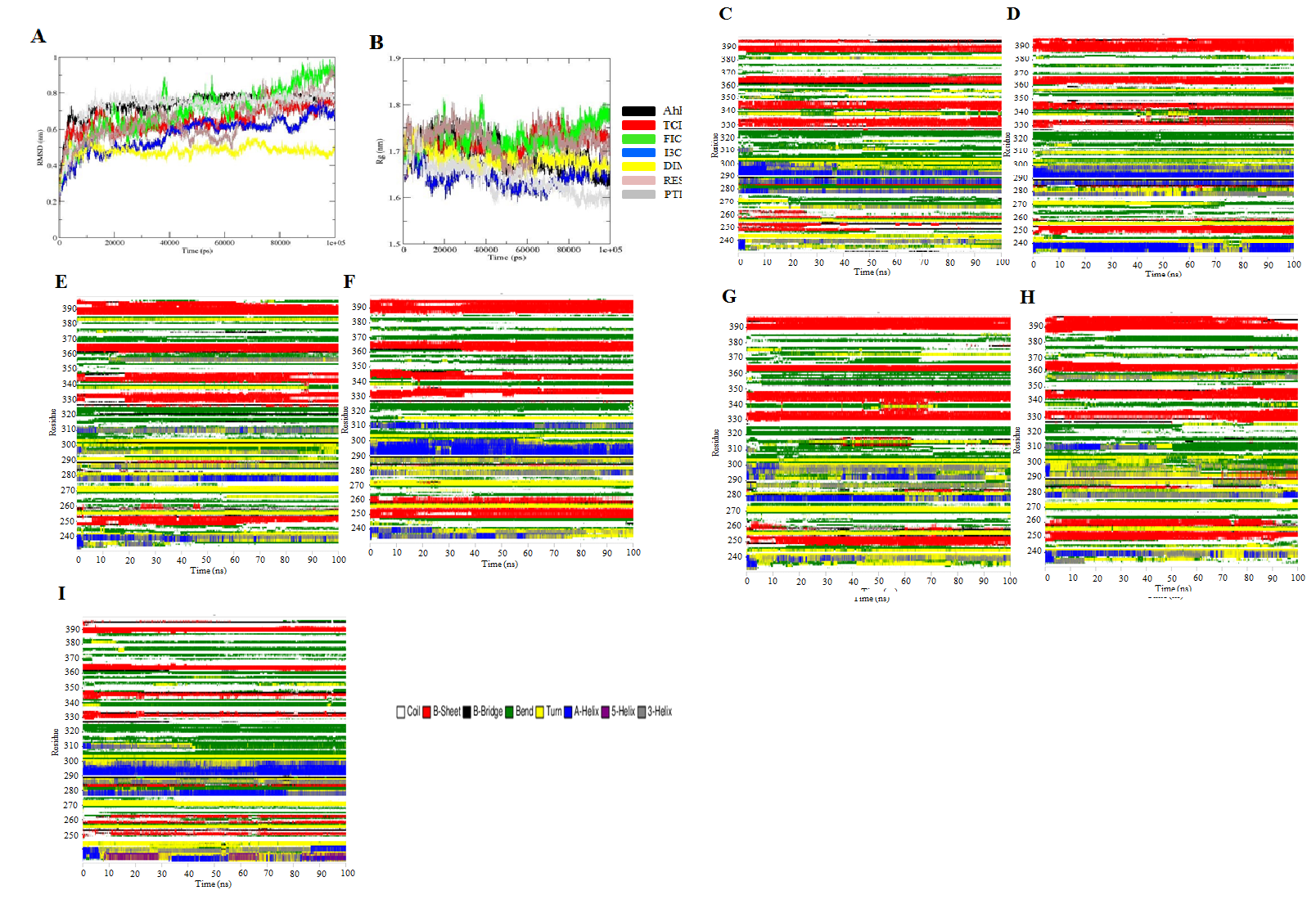

Supplement: Supplementary file 5 — Conformational changes. A Represent the Cα RMSD values of the AhRLBD and its ligands B Represents the Cα Rg of the AhRLBD and its ligands. Time evolution of Secondary structure elements during the 100 ns MDS for C AhR LBD D AhRLBD-TCDD complex E AhRLBD-FICZ F AhRLBD-I3C G AhRLBD-DIM H AhRLBD-RES I AhRLBD-PTL. The color scale at the bottom of each plot represents the secondary structure elements classified based on DSSP classification of each secondary structure element. (TIF 1805 kb) [file 12900_2018_95_MOESM5_ESM.tif]

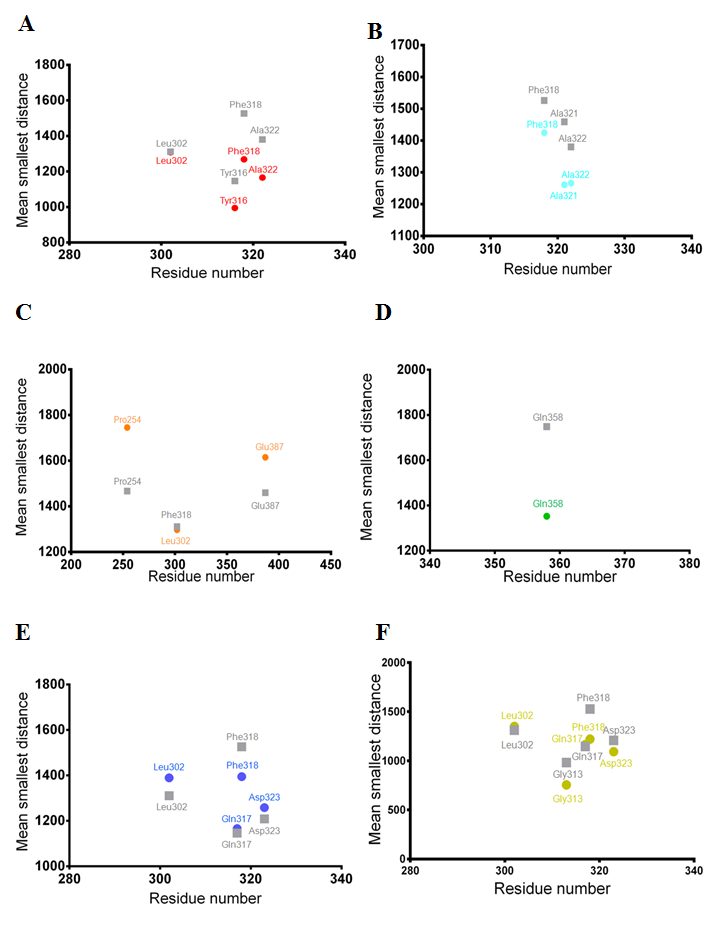

Supplement: Supplementary file 9 — Mean smallest distance for the interacting residues. A Represents the interacting residues in AhRLBD and AhRLBD-TCDD complex B Represents the interacting residues in AhRLBD and AhRLBD-FICZ complex C Represents the interacting residues in AhRLBD and AhRLBD-I3C complex D Represents the interacting residues in AhRLBD and AhRLBD-DIM complex E Represents the interacting residues in AhRLBD and AhRLBD-RES complex F Represents the interacting residues in AhRLBD and AhRLBD-PTL complex. Residues in AhRLBD shown in grey color; AhRLBD-TCDD complex residues shown in red color; AhRLBD-FICZ complex residues shown in cyan color; AhRLBD-I3C complex residues shown in orange color; AhRLBD-DIM complex residues shown in green color; AhRLBD-RES complex residues shown in violet color; AhRLBD-PTL complex residues shown in pale yellow color. (TIF 119 kb) [file 12900_2018_95_MOESM9_ESM.tif]
