# Supplementary material for: Comparative analysis of interactions between aryl hydrocarbon receptor ligand binding domain with its ligands: a computational study
Source: BMC Struct Biol. 2018 Dec 6;18:15. doi: 10.1186/s12900-018-0095-2 (PMC6282305; doi:10.1186/s12900-018-0095-2)
Supplement: Supplementary file 4 — Summary of interacting amino acid residues with the various AhR ligands under study upon docking in each of the predicted binding site. Residues forming H-bonds are shown in bolded italics. (DOCX 14 kb) [file 12900_2018_95_MOESM4_ESM.docx]

**Additional file 4. Summary of interacting amino acid residues with the various AhR ligands under study upon docking in each of the predicted binding site.** Residues forming H-bonds are shown in bolded italics

| **Structure based binding pocket** | |
| --- | --- |
| **Ligand** | **Interacting residues** |
| TCDD | ***GLY298***, ***ASP295*** |
| FICZ | ***TYR304***, ***GLU306***, ***CYS294*** |
| I3C | ***TYR304***, ***ASP295*** |
| DIM | TYR304, CYS294 |
| RES | ***GLU306***, TYR304 |
| PTL | ***CYS327***, ***TYR304***, ***GLU329***, ***ASP295*** |
| **Blind docking binding pocket** | |
| **Ligand** | **Interacting residues** |
| TCDD | LEU302, TYR316, ***PHE318***, ***ALA322*** |
| FICZ | ***PHE318***, ***ALA321*, *ALA322*** |
| I3C | ***TRP356***, ***GLN358*** |
| DIM | ***GLN358*** |
| RES | ***LEU302, GLN317,*** PHE318, ***ASP323*** |
| PTL | LEU302, ***GLY313***, ***GLN317***, PHE318, ***ASP323*** |
| **3DLigandSite docking binding pocket** | |
| **Ligand** | **Interacting residues** |
| TCDD | TYR304, ***ASP323*, *MET324***, ***CYS327*** |
| FICZ | ***HIS326***, ***CYS327***, ***GLU329*** |
| I3C | ***PRO254, LEU302, GLU387*** |
| DIM | ***HIS326*** |
| RES | ***ASP295***, ***TYR304***, ***CYS327***, ***GLU329*** |
| PTL | ***CYS294, ASP295*, *GLY298***, ***TYR304*, *CYS327, GLU329*** |
