# Supplementary material for: Comparative analysis of interactions between aryl hydrocarbon receptor ligand binding domain with its ligands: a computational study
Source: BMC Struct Biol. 2018 Dec 6;18:15. doi: 10.1186/s12900-018-0095-2 (PMC6282305; doi:10.1186/s12900-018-0095-2)
Supplement: Supplementary file 6 — Average Summary of interacting amino acid residues with the various AhR ligands under study upon docking in each of the predicted binding site. (DOCX 13 kb) [file 12900_2018_95_MOESM6_ESM.docx]

**Additional file 6.** Average Summary of interacting amino acid residues with the various AhR ligands under study upon docking in each of the predicted binding site

|  | **Backbone RMSD (nm)** | **C-alpha RMSD (nm)** | **Backbone Rg (nm)** | **C-alpha Rg (nm)** |
| --- | --- | --- | --- | --- |
| **AhR LBD** | 0.73 ± 0.0 | 0.74 ± 0.0 | 1.69 ± 0.0 | 1.69 ± 0.0 |
| **Ahr LBD-TCDD** | 0.65 ± 0.0 | 0.65 ± 0.0 | 1.71 ± 0.0 | 1.72 ± 0.0 |
| **Ahr LBD-FICZ** | 0.70 ± 0.1 | 0.70 ± 0.1 | 1.72 ± 0.0 | 1.73 ± 0.0 |
| **Ahr LBD-I3C** | 0.56 ± 0.0 | 0.57 ± 0.0 | 1.64 ± 0.0 | 1.64 ± 0.0 |
| **Ahr LBD-DIM** | 0.47 ± 0.0 | 0.48 ± 0.0 | 1.68 ± 0.0 | 1.68 ± 0.0 |
| **Ahr LBD-RES** | 0.64 ± 0.1 | 0.64 ± 0.1 | 1.72 ± 0.0 | 1.72 ± 0.0 |
| **Ahr LBD-PTL** | 0.73 ± 0.0 | 0.73 ± 0.0 | 1.63 ± 0.0 | 1.63 ± 0.0 |
