# Supplementary material for: Comparative analysis of interactions between aryl hydrocarbon receptor ligand binding domain with its ligands: a computational study
Source: BMC Struct Biol. 2018 Dec 6;18:15. doi: 10.1186/s12900-018-0095-2 (PMC6282305; doi:10.1186/s12900-018-0095-2)
Supplement: Supplementary file 7 — Calculated binding energies using MD-MM/PBSA or direct MM/PBSA for the six AhR-ligand complexes. (DOCX 13 kb) [file 12900_2018_95_MOESM7_ESM.docx]

**Additional file 7.** Calculated binding energies using MD-MM/PBSA or direct MM/PBSA for the six AhR-ligand complexes

| **AhR-ligand complex** | **ΔE_elec_** | **ΔE_vdW_** | **ΔG_pol_** | **ΔG_SASA_** | **ΔG_binding_** |
| --- | --- | --- | --- | --- | --- |
| TCDD | -8.898 ± 9.0 | -76.098 ± 50.8 | -0.215 ± 40.3 | -7.163 ± 4.7 | -92.374 ± 78.3 |
| FICZ | -5.746 ± 5.9 | -76.099 ± 44.4 | 27.585 ± 21.3 | -8.081 ± 4.6 | -62.342 ± 37.6 |
| I3C | -2.901 ± 6.9 | -13.715 ± 21.2 | 0.195 ± 27.1 | -2.035 ± 3.2 | -18.456 ± 26.5 |
| DIM | -9.439 ± 9.6 | -60.803 ± 33.8 | 21.728 ± 29.8 | -7.638 ± 4.0 | -56.153 ± 27.9 |
| RES | -7.861 ± 17.4 | -19.482 ± 42.1 | -0.215 ± 40.3 | -2.114 ± 4.7 | -29.672 ± 28.6 |
| PTL | -16.310 ± 13.9 | -76.391 ± 52.7 | 44.674 ± 36.9 | -8.024 ± 5.5 | -56.051 ± 38.2 |

All values are given in average  ±  S.D.

ΔE_elec_: Electrostatic energy; ΔE_vdW_: Van der Waal energy; ΔG_pol_: Polar solvation energy; ΔG_SASA_: Non-Polar solvation energy calculated based on SASA; ΔG_binding_: Binding free energy
