# Supplementary material for: Comparative analysis of interactions between aryl hydrocarbon receptor ligand binding domain with its ligands: a computational study
Source: BMC Struct Biol. 2018 Dec 6;18:15. doi: 10.1186/s12900-018-0095-2 (PMC6282305; doi:10.1186/s12900-018-0095-2)
Supplement: Supplementary file 8 — Percentage of secondary structure elements during the 100 ns simulation. The percentages were calculated using the DSSP program in Gromacs. (DOCX 14 kb) [file 12900_2018_95_MOESM8_ESM.docx]

**Additional file 8. Percentage of secondary structure elements during the 100 ns simulation.** The percentages were calculated using the DSSP program in Gromacs

|  | **Structure** | **Coil** | **B-Sheet** | **B-Bridge** | **Bend** | **Turn** | **A-Helix** | **5-Helix** | **3-Helix** |
| --- | --- | --- | --- | --- | --- | --- | --- | --- | --- |
| **AhR** | 0.38 | 0.33 | 0.17 | 0.03 | 0.24 | 0.13 | 0.05 | 0.00 | 0.05 |
| **AhRLBD-TCDD** | 0.40 | 0.34 | 0.17 | 0.03 | 0.23 | 0.12 | 0.09 | 0.00 | 0.03 |
| **AhRLBD-FICZ** | 0.41 | 0.32 | 0.18 | 0.04 | 0.22 | 0.15 | 0.03 | 0.00 | 0.05 |
| **AhRLBD-I3C** | 0.42 | 0.33 | 0.20 | 0.02 | 0.21 | 0.12 | 0.08 | 0.04 | - |
| **AhRLBD-DIM** | 0.40 | 0.31 | 0.19 | 0.02 | 0.25 | 0.15 | 0.04 | 0.00 | 0.04 |
| **AhRLBD-RES** | 0.40 | 0.33 | 0.21 | 0.02 | 0.22 | 0.15 | 0.02 | 0.00 | 0.05 |
| **AhRLBD-PTL** | 0.34 | 0.36 | 0.11 | 0.04 | 0.25 | 0.12 | 0.08 | 0.01 | 0.04 |

where Structure = A-Helix + B-Sheet + B-Bridge + Turn
